# Localization of the ATP-binding cassette (ABC) transport proteins PfMRP1, PfMRP2, and PfMDR5 at the *Plasmodium falciparum *plasma membrane

**DOI:** 10.1186/1475-2875-8-205

**Published:** 2009-08-28

**Authors:** Reginald A Kavishe, Jeroen MW van den Heuvel, Marga van de Vegte-Bolmer, Adrian JF Luty, Frans GM Russel, Jan B Koenderink

**Affiliations:** 1 Department of Pharmacology and Toxicology 149, Radboud University Nijmegen Medical Centre, P.O. Box 9101, 6500 HB Nijmegen, the Netherlands; 2 Department of Medical Microbiology 268, Radboud University Nijmegen Medical Centre, P.O. Box 9101, 6500 HB Nijmegen, the Netherlands; 3 Kilimanjaro Christian Medical College of Tumaini University, P. O. Box 2240, Moshi, Tanzania

## Abstract

**Background:**

The spread of drug resistance has been a major obstacle to the control of malaria. The mechanisms underlying drug resistance in malaria seem to be complex and multigenic. The current literature on multiple drug resistance against anti-malarials has documented PfMDR1, an ATP-binding cassette (ABC) protein, as an important determinant of resistance. In the *Plasmodium falciparum *genome, there are several ABC transporters some of which could be putative drug transporting proteins. In order to understand the molecular mechanisms underlying drug resistance, characterization of these transporters is essential. The aim of this study was to characterize and localize putative ABC transporters.

**Methods:**

In the plasmoDB database, 16 members of the *P. falciparum *ABC family can be identified, 11 of which are putative transport proteins. A phylogenetic analysis of the aligned NBDs of the PfABC genes was performed. Antibodies against PfMRP1 (PfABCC1), PfMRP2 (PfABCC2), and PfMDR5 (PfABCB5) were generated, affinity purified and used in immunocytochemistry to localize the proteins in the asexual stages of the parasite.

**Results:**

The ABC family members of *P. falciparum *were categorized into subfamilies. The ABC B subfamily was the largest and contained seven members. Other family members that could be involved in drug transport are PfABCC1, PfABCC2, PfABCG1, and PfABCI3. The expression and localization of three ABC transport proteins was determined. PfMRP1, PfMRP2, and PfMDR5 are localized to the plasma membrane in all asexual stages of the parasite.

**Conclusion:**

In conclusion, 11 of the 16 ABC proteins in the *P. falciparum *genome are putative transport proteins, some of which might be involved in drug resistance. Moreover, it was demonstrated that three of these proteins are expressed on the parasite's plasma membrane.

## Background

**Figure 1:**
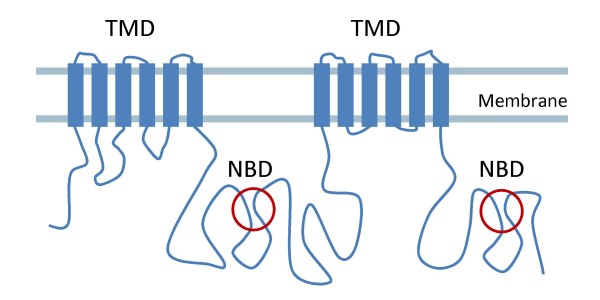
**Typical structure of an ABC full transporter**. The TMDs each contain 6 trans-membrane (TM) segments and the NBDs contain the Walker A and Walker B motifs. ABC half transporters consists of one TMD and one NBD that upon translation combine to form a functional unit.

Drug resistance is a major problem in malaria. Today only a limited number of effective anti-malarials is available. An important reason for therapeutic failure in malaria treatment could be that drugs do not reach their target sites, due to active extrusion by the parasite. The transport proteins responsible for this type of resistance are so-called multidrug resistance proteins (MDR/MRP), most of which belong to the superfamily of ATP binding cassette (ABC) proteins, one of the largest protein families. Many of these plasma membrane proteins actively pump out a wide range of structurally and functionally diverse amphipathic drugs, thereby decreasing the intracellular drug accumulation and resulting in drug resistance [1,2]. The structure of a typical ABC transporter consists of six trans-membrane segments that form a trans-membrane domain (TMD) and the Walker A and Walker B motifs that form a nucleotide binding domain (NBD) (Figure 1). ABC transporters are either encoded as full transporters (TMD-NBD-TMD-NBD) or as half transporters (TMD-NBD) that upon translation combine to form a functional unit. Apart from their normal physiological role, ABC transporters are involved in various diseases either by a mutation or through an altered mode of their expression [3,4].

Several polymorphisms and an increased copy number of the *Plasmodium falciparum *MDR1 have been associated with drug resistance [5]. Heterologous expression data indeed show that PfMDR1-mediated anti-malarial drug transport is affected by some of these polymorphisms [6,7]. The other member of the PfABC B family that has been characterized is the half transporter PfMDR2 that confers heavy metal resistance to *P. falciparum *and most likely is not involved in drug resistance [8]. Finally, PfMRP1 polymorphisms have been associated with drug resistance and recently it has been shown that PfMRP1 transports anti-malarial drugs and glutathione [9]. PfMDR1 has been detected in the membrane of the food vacuole [10], which is in contrast to PfMRP1 that has been immunelocalized in the plasma membrane of the parasite [9,11]. In previous studies the *P. falciparum *ABC family members were identified [12,13] and categorized [14,15]. In this study, the *P. falciparum *ABC transporter family has also been categorized and the database sequence of PfMRP1, PfMRP2, and PfMDR5 was confirmed. Moreover, with immunocytochemistry techniques it was shown that all three transporters are probably present in the plasma membrane of the parasite during the asexual erythrocytic stages.

## Methods

### Parasite culture, extraction and DNA isolation

NF54 (Amsterdam airport) strain of *P. falciparum *was cultured as described by Ponnudurai *et al *[16]. The culture medium [17] was changed twice daily. Infected blood culture was centrifuged at 1000 g for 5 minutes to collect RBCs. After washing twice with PBS the cells were resuspended in 0.05% saponin in PBS and incubated at 37°C for 30 minutes. Then the sample was centrifuged at 2000 g for 10 minutes to collect the parasites. Parasite cells were washed twice with PBS and genomic DNA was isolated using the QIAamp DNA mini kit (Qiagen, Venlo, The Netherlands) following the cultured cells protocol as directed by the manufacturer. RNA was isolated using standard Trizol (guanidinium isothiocyanate) method. Briefly, 500 μl Trizol reagent (Invitrogen) was added to the parasite pellet followed with 2.5 μl glycogen solution (18 mg/ml), the mixture was homogenized with pipette and incubated on ice for 5 min 50 μl chloroform was added and after shaking thoroughly the mixture was incubated on ice for 10 min. Centrifugation at 13,000 g for 15 min at 4°C separated the mixture into upper aqueous and lower chloroform phases. The aqueous phase which contains RNA was transferred into a fresh tube and 240 μl isopropyl alcohol was added, mixed by vortex and incubated on ice for 10 min prior to centrifugation at 13,000 g for 20 min at 4°C. The supernatant was discarded and the RNA pellet was air-dried and dissolved in 20 μl DEPC treated water. RNA was stored at -20°C until use.

### Sequencing of PfMRP1, PfMRP2, and PfMDR5

cDNA was synthesized with superscript-II reverse transcriptase and oligo-dT primers (Invitrogen). PCR for the ABC genes was performed on cDNA using Takara LA Taq. PCR protocol was followed as outlined on Takara protocol except that annealing was done at 58°C for 30 sec, extension at 62°C for 10 min, 25 cycles with final extension 72°C for 5 minutes. The PCR product was PEG-purified and cloned into pDONR-221 entry-vector using BP clonase kit (Invitrogen) following manufacturer's instructions. Sequencing was performed using ABI3730 analyzer (Applied biosystems Inc.).

### Antibodies

PfMRP1 polyclonal antibody was raised in rabbits against the specific peptides ^215^CSNNNHLQNPDAFY^228 ^and ^1420^YASGIIKLYKEKNYV^1434 ^as described by Klokousas *et al *[11] (Eurogentec, Belgium), whereas the PfMDR5 polyclonal antibody was designed and produced in rabbits (EZ Biolab, USA) against the peptide ^730^CQSTKYNSQCYQKNK^744 ^in the cytosolic loop of the protein. For PfMRP2 a GST-fusion peptide was produced in *Escherichia coli *using pGEX-3X vector. This peptide corresponds to a 70 amino acid long region within the cytosolic loop of PfMRP2 represented by the sequence: LHYEGNLVDYIKKNNIVVKEDIVQTNKQCEKKSLTNEQVKSMLSLNEDWNYMHRVKKKSITQKETTKNYD. The peptide was extracted and purified from *E. coli *using glutathione-agarose beads (BD Biosciences). 2 ml of the purified peptide was subcutaneously injected into rabbits followed with a 1^st ^and 2^nd ^boost on day 21 and 42, respectively. Pre-immune serum was taken on day zero and after 63 days serum was collected. For immuno-localization experiments, the polyclonal sera for PfMRP1 and PFMDR5 were affinity purified using Sulfolink immobilization kit for peptides (Thermo scientific, USA) while for PfMRP2 the Affigel-15 kit (Bio-Rad Laboratories) was used as described by the manufacturers.

### Immuno-localization

Infected RBCs were fixed in 4% paraformaldehyde for 10 minutes at room temperature then washed once with PBS. The cells were applied on poly-L-lysine coated slide cover slips (12 mm, Menzel GmbH & co KG, Germany) and air dried for 5 – 10 minutes. Cover slips were quenched with 0.15% glycine in 0.5% PBS-tween20 (PBST) for 10 minutes at room temp followed by washing twice with PBST and incubation with 0.1% tritonX100 (BDH chemicals) for 45 minutes at room temp. After washing once with PBST, blocking was done with normal goat serum (Zymed, USA) for 1 hr at room temp followed with washing once with PBST. Cover slips were then incubated with primary antibody: rabbit polyclonals and mouse anti-PfERC (MR4, MRA-87 Pf39 mouse antiserum, deposited by TJ Templeton) or mouse anti-glycophorin A monoclonals (Caltag laboratories, Invitrogen) at 4°C overnight followed with three times washing each for 20 minutes with PBST at room temperature. The secondary antibodies Alexa 594 goat anti-rabbit and Alexa 488 goat anti-mouse monoclonals (Molecular probes, The Netherlands) were applied on the cover slips for 2 hrs at room temperature. After rinsing two times with PBST, the cover slips were incubated for 30 minutes with DAPI followed with 2 × 20 minutes washing with PBST at room temperature. The cover slips were then briefly air-dried in the dark and mounted on microscope slides (Menzel GmbH & co KG, Germany) using Dako fluorescent mounting medium (Dako North America Inc., Carpinteria USA). Slides were imaged using a confocal microscope (Olympus FV1000). Image J free software version 1.43 was used to process images.

## Results

### Identification and characterization of ABC transport proteins

**Figure 2:**
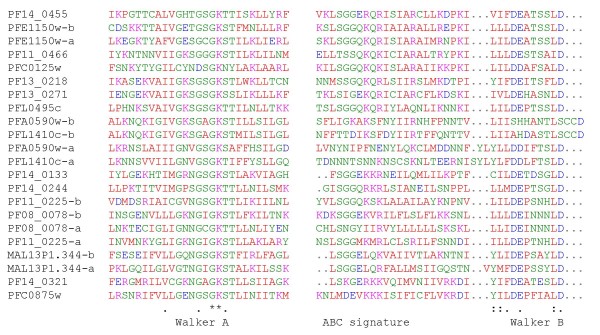
**NBD alignment of *P. falciparum *ABC superfamily**. The figure shows a part of the with ClustalW2 aligned sequence with Walker A (GxxGxGKST or [AG]xxxxGK [ST]), ABC signature sequence (LSGGQ), and Walker B (hhhhDEPT or DExxxxxD). x is any amino acid whereas h is a hydrophobic amino acid.

**Figure 3:**
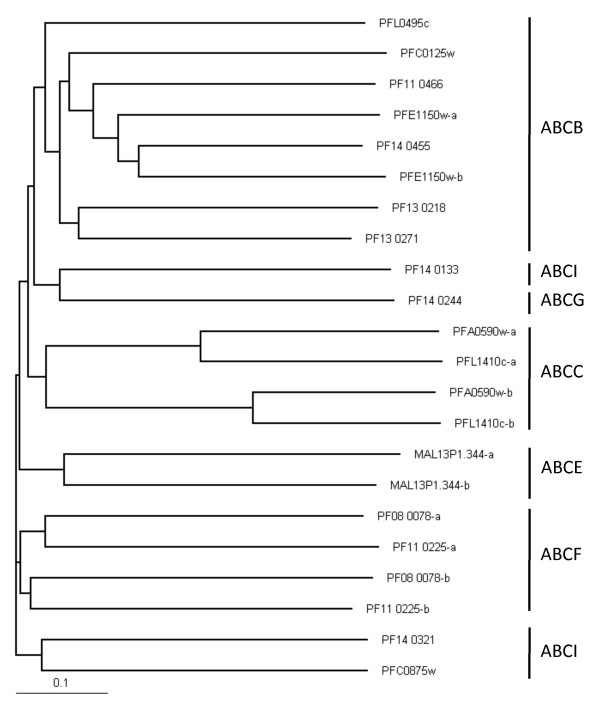
**Phylogram of the *P. falciparum *ABC superfamily**. The NBDs were aligned and the phylogenetic data was imported in TreeView. In combination with blast searches the ABC transport proteins were categorized in subfamilies.

In the plasmoDB database, a PFAM (PF00005:ABC_tran ABC transporter) search for ABC transport proteins in *P. falciparum *resulted in 16 hits. PfMDR1 (PFE1150w), PfMDR2 (PF14_0455), and PfMRP1 (PFA0590w) are hits that have been described in literature by several groups [8-10], but the other family members have not been the subject of investigation yet. In this study it was determined if the family members are full (two NBDs) or half transporters (one NBD). Next the NBD (30 amino acids before Walker A until Walker B) were aligned with ClustalW2 [18]. Part of the aligned sequence with the Walker A (GxxGxGKST or [AG]xxxxGK [ST]), the ABC signature sequence (LSGGQ), and Walker B (hhhhDEPT or DExxxxxD) are shown in Figure 2. The phylogenetic data was imported in TreeView [19] and visualized as a phylogram in Figure 3. A blast search against human proteins clearly shows that there are no members of the A subfamily. However, *P. falciparum *contains 11 putative transport proteins containing TMDs that belong to the B, C, G and I subfamilies. In addition, there are five ABC family members that do not contain a TMD. Three of these belong to the E and F subfamilies, whereas the other two members were classified in the I subfamily that was introduced for the plant ABC proteins recently [20]. The numbering within each subfamily is shown in Table 1 and was done according to the existing chronological numbering (PfMDR1, PfMDR2, PfMRP1, and PfMRP2). Moreover, neighbouring ABC members in the phylogenetic tree obtained sequential numbering. The numbers do not carry additional information, such as a relationship with ABC transporters of other species. The domain organization was established by analysis of the sequences with SMART [21].

**Table 1:** The *Plasmodium falciparum *ABC superfamily

**Subfamily**	**Gene identification**		**Domain organisation**	**Amino acids**
**ABCB**				
ABCB1	PFE1150w	PfMDR1	(MSD-NBD)_2_	1419
ABCB2	PF14_0455	PfMDR2	MSD-NBD	1024
ABCB3	PF11_0466		MSD-NBD	872
ABCB4	PFC0125w		MSD-NBD	1365
ABCB5	PF13_0218		MSD-NBD	925
ABCB6	PF13_0271		MSD-NBD	1049
ABCB7	PFL0495c		MSD-NBD	855

**ABCC**				
ABCC1	PFA0590w	PfMRP1	(MSD-NBD)_2_	1822
ABCC2	PFL1410c	PfMRP2	(MSD-NBD)_2_	2108

**ABCE**				
ABCE1	MAL13 P1.344		(NBD)_2_	619

**ABCF**				
ABCF1	PF11_0225		(NBD)_2_	815
ABCF2	pfGCN20 PF08_0078		(NBD)_2_	1419

**ABCG**				
ABCG1	PF14_0244		NBD-MSD	660

**ABCI**				
ABCI1	PF14_0133		NBD	347
ABCI2	PF14_0321		NBD	171
ABCI3	PFC0875w		MSD-NBD-MSD	3133

This study focuses on PfMRP1 (PFA0590w), PfMRP2 (PFL1410c), and PfMDR5 (PF13_0218), because their antibody generation was successful. PfMRP1 and PfMRP2 are both full transporters that belong to the ABC C family, whereas PfMDR5 is a half transporter that belongs to the ABC B family. For these three transporters, the trans-membrane segments were determined with TMHMM [22]. Some of the trans-membrane segments were not predicted, but weak probabilities of TM helices were assigned as such in order to obtain the correct ABC trans-membrane domains with six trans-membrane segments (Table 2). PfMRP1, PfMRP2, and PfMDR5 genes were sequenced and the results matched the published data and no polymorphisms were observed.

**Table 2:** Trans-membrane segments of PfMRP1, PfMRP2, and PfMDR5.

**TM**	**PfMRP1**	**PfMRP2**	**PfMDR5**
1	121–143	133–155	*88–111*
2	175–197	180–202	*143–163*
3	318–340	402–424	181–203
4	350–372	434–456	270–292
5	426–448	517–539	365–387
6	*466–488*	*554–575*	399–417
7	1158–1180	1430–1452	
8	1218–1240	1473–1495	
9	1250–1272	1510–1532	
10	1293–1310	1553–1570	
11	1314–1333	1574–1593	
12	1392–1414	1654–1676	

The TM segments were predicted with TMHMM. TM segments in italic were weak probabilities.

### Localization of PfMRP1, PfMRP2, and PfMDR5

Polyclonal antibodies were generated by immunization of rabbits with specific peptides for the *P. falciparum *ABC proteins. Blast searches with the peptides showed no relevant specific hits with other proteins. To minimize aspecific binding immuno-purified antibodies against the peptide were used for immuno-localization studies. The glycophorin-A antibody was used to stain erythrocyte membranes thus contrasting them from parasite cells while the PfERC antibody, which binds to an intracellular calcium binding protein located on the endoplasmic reticulum, was used to determine intracellular localization of the pfABCs.

**Figure 4:**
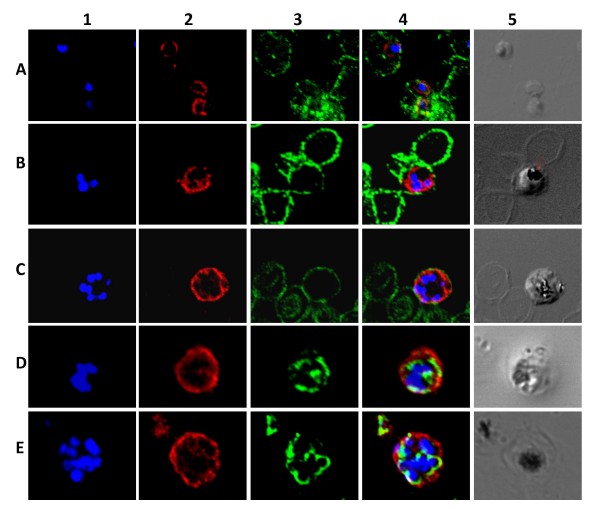
**Localization of PfMRP1 within the asexual stages of *P. falciparum***. In column 1 blue (DAPI) shows the nuclear staining. In column 2 red shows PfMRP1 staining. In column 3 green shows glycophorin A (A, B, and C) or PfERC (D and E) staining. Column 4 shows the merge of column 1–3. Finally, column 5 shows the differential interference contrast image. Represented in rows: A, rings; B, early schizont; C, schizont; D, trophozoite; E, schizont and ring. The arrow indicates the food vacuole.

PfMRP1 localized to the parasites plasma membrane and no signal was observed on the erythrocyte membrane or in the region around the food vacuole. In the differential interface contrast image the food vacuole can be recognized from its dark-spot appearance (presence of haemozoin crystals). These results are consistent with those reported in a recent study [9] in which plasma membrane localization of PfMRP1 was reported also, with possible intracellular expression. The results in this study show the signal to be intense on the outside, forming a clear rim in what can be interpreted as the parasite's plasma membrane especially in the ring and trophozoite stages (Figures 4A–D). More clearly cytoplasmic staining was evident in the schizont stages (Figures 4B, C, and 4E), interpreted as plasma membrane partitions of the dividing parasites. Unfortunately, due to their small size detailed high magnification imaging of the non-dividing stages such as rings and young trophozoites was limited by the light resolution power of the microscope. PfMRP1 did not co-localize with PfERC, thus suggesting the absence of PfMRP1 on the endoplasmic reticulum membrane.

**Figure 5:**
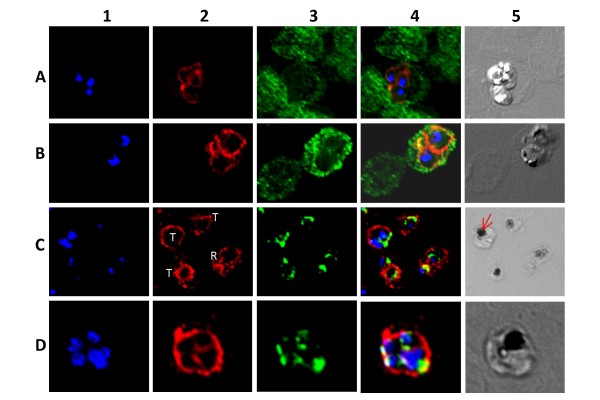
**Localization of PfMRP2 within the asexual stages of *P. falciparum***. In column 1 blue (DAPI) shows the nuclear staining. In column 2 red shows PfMRP2 staining. In column 3 green shows glycophorin-A (A and B) or PfERC (C and D) staining. Column 4 shows the merge of column 1–3. Finally, column 5 shows the differential interference contrast image. Represented in rows: A, multiple infected erythrocyte: rings; B, double infected erythrocyte: trophozoites; C, three trophs (T) and two rings (R); D, early schizont. Arrow indicates food vacuole.

For PfMRP2 specific staining was observed on the parasite's plasma membrane (Figure 5, column 2). Low intensity signal was occasionally observed intracellularly especially in early schizont stages (Figure 5, D2) which can be considered to be plasma membrane partitions as the parasite undergoes cytokinesis. This is especially so as each of these partitions enclose a nucleus as clearly stained by DAPI (Figure 5, D4). PfMRP2 did not co-localize with PfERC in the endoplasmic reticulum (Figure 5C and 5D) and no staining was observed in the region presumed to be the food vacuole (shown by arrow in Figure 5, C5).

**Figure 6:**
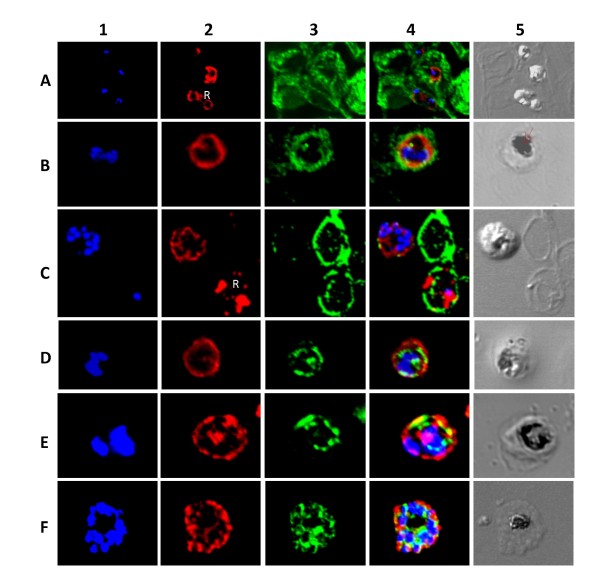
**Localization of PfMDR5 within the asexual stages of *P. falciparum***. In column 1 blue (DAPI) shows the nuclear staining. In column 2 red shows PfMDR5 staining. In column 3 green shows glycophorin-A (A, B, and C) or PfERC (D, E, and F) staining. Column 4 shows the merge of column 1–3. Finally, column 5 shows the differential interference contrast image. Represented in rows: A, rings; B, trophozoite; C, schizont and ring (R); D, early trophozoite; E, dividing trophozoite; F, schizont. Arrow indicates food vacuole

PfMDR5 was clearly observed to localize to the surface of the parasite in all asexual stages (Figure 6). Trophozoite stages sometimes revealed a less intense intracellular staining around the food vacuole (Figure 6, B5 and D5). This pattern of staining though sometimes strong, was not consistent in the many slides scanned. Instead the staining was sometimes seen around the nuclei of dividing trophozoites, which may also suggest these to be plasma membrane partitions as the trophozoite divides. Moreover, in the schizont stages the region covered by the food vacuole did not show any obvious staining with the PfMDR5 antibody, instead clear partitions were seen separating each of the individual nuclei (Figure 6, 2C). In comparison with the endoplasmic reticulum marker PfERC, which localized as bright staining patches within the parasite, PfMDR5 localized differently, forming a rim outside the PfERC staining in all stages (Figure 6D–F) (ring stages not shown). Therefore, it is concluded that PfMDR5 is primarily expressed on the plasma membrane.

## Discussion

In the plasmoDB database 16 members of the *P. falciparum *ABC family can be identified, 11 of which could be putative drug efflux transporters. A phylogenetic analysis of the PfABC genes was performed and the genes were categorized into seven subfamily groups. Three ABC family members were analysed in more detail and their expression in all erythrocytic stages as well as their localization at the plasma membrane of the parasite was demonstrated.

Development of resistance against newly introduced anti-malarials can be rapid especially in malaria endemic regions where drug pressure is high. For instance mefloquine was introduced in Thailand in 1984 as a very potent drug against multidrug resistant malaria, but six years later significant resistance had developed [23]. Multiple studies have demonstrated the role of PfMDR1 polymorphisms in multidrug resistant malaria. It was shown that the anti-malarial drugs halofantrine, quinine and chloroquine are transported by PfMDR1 [7]. Moreover, polymorphisms within PfMDR1 alter the substrate specificity for these anti-malarial drugs [7]. Recent association studies have linked single nucleotide polymorphisms on PfMRP1 with reduced parasite response to anti-malarial drugs [24,25] and expression of both PfMRP1 and PfMRP2 were up-regulated by mefloquine and chloroquine in laboratory cultures of both drug sensitive and resistant strains [26]. This indicates that ABC transporters play a role in malaria chemotherapy [27]. The knowledge that other PfABC family members could be capable of drug transport indicates that it is likely that some of these transport proteins might play a role in emerging drug resistance. Further identification and characterization of these ABC transporters will provide information on their putative role in resistance and may provide novel targets to control and perhaps prevent spread of resistance against other efficacious drugs.

The PFAM search in plasmoDB resulted in 16 ABC family members that were also identified and catagorized by others [12-15]. ABC A subfamily members were not observed, but these seem to be absent in all Apicomplexa. The ABC B subfamily consists of seven members of which only PfMDR1 is a full transporter. The C subfamily contains two full transporters, whereas the G subfamily contains one half transporter. Next to these ABC family members there is one additional member that contains a TMD. As this member does not belong to the existing subfamilies, it was placed in the I family that was proposed by Verrier *et al *[20] to harbour "orphan" ABC components. Five ABC family members that do not contain TMDs are located in E, F, and I subfamilies.

Recently, Gangwar and colleagues [14] analysed the ABC family members of *P. falciparum*, their phylogenetic tree was, however, not based on an alignment of the conserved NDB regions but on the whole protein, which results in a phylogenetic tree where the subfamilies are not clustered. Moreover, they did not include ABCB4 (PFC0125w) in their analyses and included PFE0450w, which is not an ABC family member but a putative chromosome condensation protein. In a very recent review by Sauvage *et al *[15] the ABC families of different protozoan parasites were listed. They clustered the ABC family members in a way similar to our approach. In this study PF08_0078 was, however, assigned in the F family according to the phylogenetic analysis of both NBDs, whereas they categorized it as "other". As they did not show a phylogenetic analysis it is not clear why this discrepancy exists. Moreover, PFC0875w (PfABCI3), that does not possess a clear ABC signature motif, but according to the PFAM search belongs to the ABC family, is not listed in their overview. In this study the existing nomenclature for PfMDR1, PfMDR2, and PfMRP1, was not changed, but the nomenclature of Verrier *et al *[20] for plant ABC proteins that was adopted from the HUGO nomenclature and discussed in several international meetings was used. This nomenclature essentially constitutes a catalogue with numbered entries, where the numbers do not necessarily carry additional information, although the subfamily assignment does indeed convey information about phylogenetic relationships [20]. The gene nomenclature recently published by Sauvage *et al *[15] is based on the nomenclature for *Toxoplasma gondii *[28], which as a consequence in *P. falciparum *resulted in the absence of ABCB2, ABCG1, ABCG2, ABCH1, and ABCH3 and the relocation of PfMDR2 to ABCB3. Such a nomenclature is, however, impractical for a group with relatively few orthologous pairings, such as the ABCs [20].

It is known that the malaria parasite can express its membrane proteins on at least four different subcellular sites: on its membrane-bound organelles, the plasma membrane, the parasitophorous vacuolar membrane and on the plasma membrane of its host erythrocyte [29,30]. The expression of PfMRP1, PfMRP2, and PfMDR5 was observed on the outside of the parasite. It is practically impossible to distinguish the plasma membrane from the parasitophorous vacuolar membrane by immunocytochemistry. However, a detailed look at the expression of all three ABC transporters shows that they are also present on membranes between parasitic nuclei in the multi-nuclear stages of development. As these are plasma membranes in the process of formation, it is concluded that all three ABC transport proteins are located on the plasma membrane in all asexual erythrocytic stages of *P. falciparum*. The localization of PfMRP1 at the plasma membrane has been shown by others [9,11]. An elegant control was shown in the recent study of Ray *et al *[9], where a *P. falciparum *knock-out of PfMRP1 was used. In this study, knock-out parasites were not available and as an additional control the antibody was affinity purified with the peptide that was used to immunize the rabbits. In addition, background staining with the erythrocytes was not observed indicating that the staining was specific.

## Conclusion

16 ABC proteins were categorized according the phylogenetic tree that was constructed from the aligned NBDs. PfMRP1, PfMRP2, and PfMDR5 were localized at the plasma membrane of the parasite throughout the asexual stages. This localization emphasizes the putative role of drug exporters of these ABC family members. Indeed, Ray *et al *[9] have shown that PfMRP1 plays a role in the efflux of glutathione, chloroquine, and quinine and contributes to parasite responses to multiple anti-malarial drugs, possibly by pumping drugs outside the parasite. PfMRP2 and PfMDR5 might have similar roles and thereby broadening the capacity of the parasite to extrude toxic compounds. Additional research is required to test this hypothesis.

## Competing interests

The authors declare that they have no competing interests.

## Authors' contributions

RAK carried out the cloning, sequencing and immuno-localization experiments and drafted the manuscript. JJMWH participated in cloning, sequencing and immuno-localization experiments. MVB carried out the parasite culture. AJFL participated in its design and supervision and helped draft the manuscript. FGMR participated in its design and supervision and helped draft the manuscript. JBK carried out the phylogenetic analysis, conceived of the study, participated in its design and coordination and helped to draft the manuscript. All authors read and approved the final manuscript.
